# Supplementary figures and images for: SLFCNet: an ultra-lightweight and efficient strawberry feature classification network
Source: PeerJ Comput Sci. 2025 Jan 2;11:e2085. doi: 10.7717/peerj-cs.2085 (PMC11784530; doi:10.7717/peerj-cs.2085)

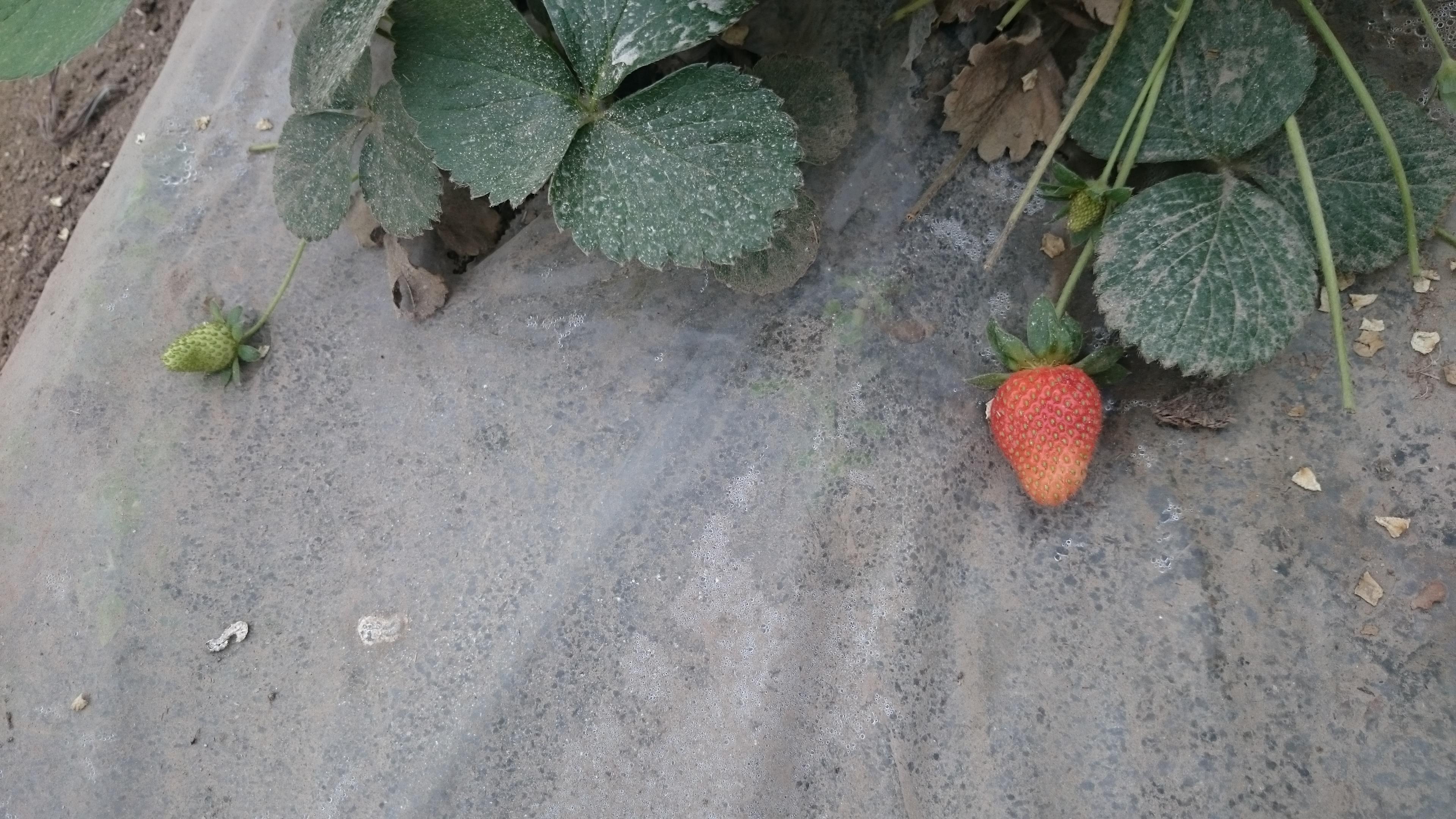

Supplement: Supplemental Information 1 [file peerj-cs-11-2085-s001.zip › ultralytics/assets/2G-3F_JPG.rf.cfe3e6769bff4e20fe07a4af04228242.jpg]

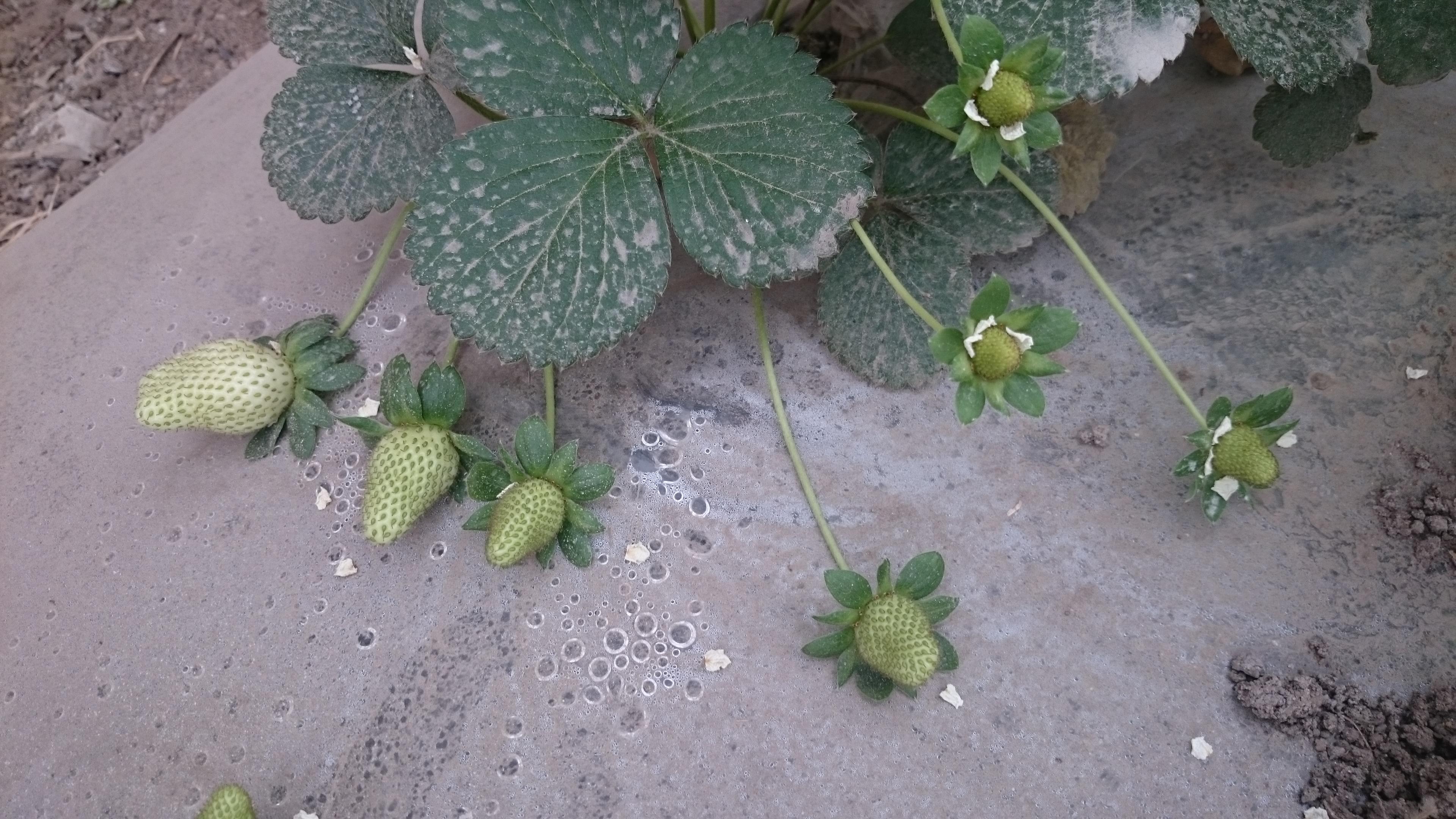

Supplement: Supplemental Information 1 [file peerj-cs-11-2085-s001.zip › ultralytics/assets/3G-7F_JPG.rf.67d4fb59a62cb5307ee47cd44fc5b089.jpg]

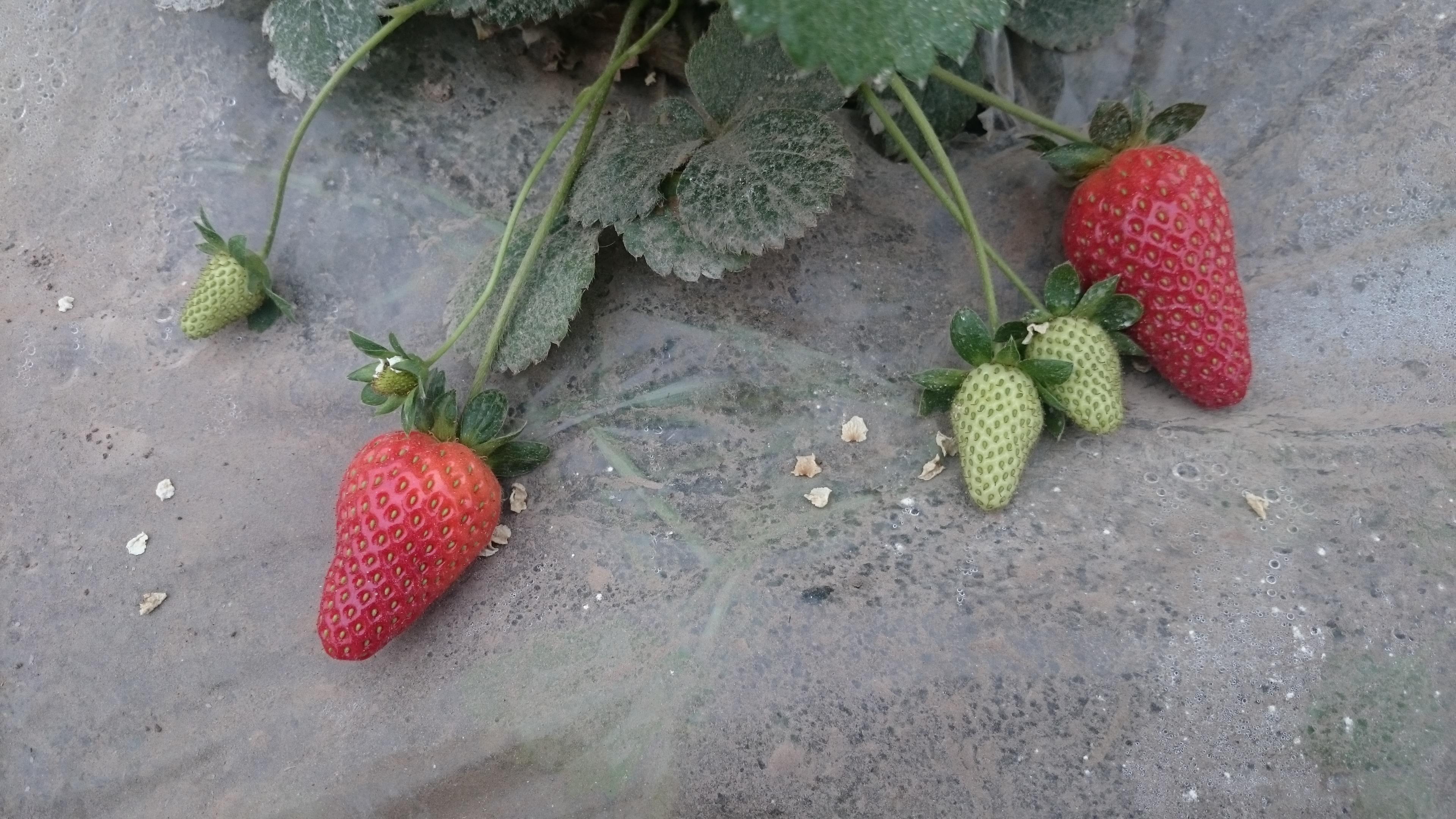

Supplement: Supplemental Information 1 [file peerj-cs-11-2085-s001.zip › ultralytics/assets/4G-6F_JPG.rf.ed5953ad8180c37f2f54f7946f7d57e8.jpg]

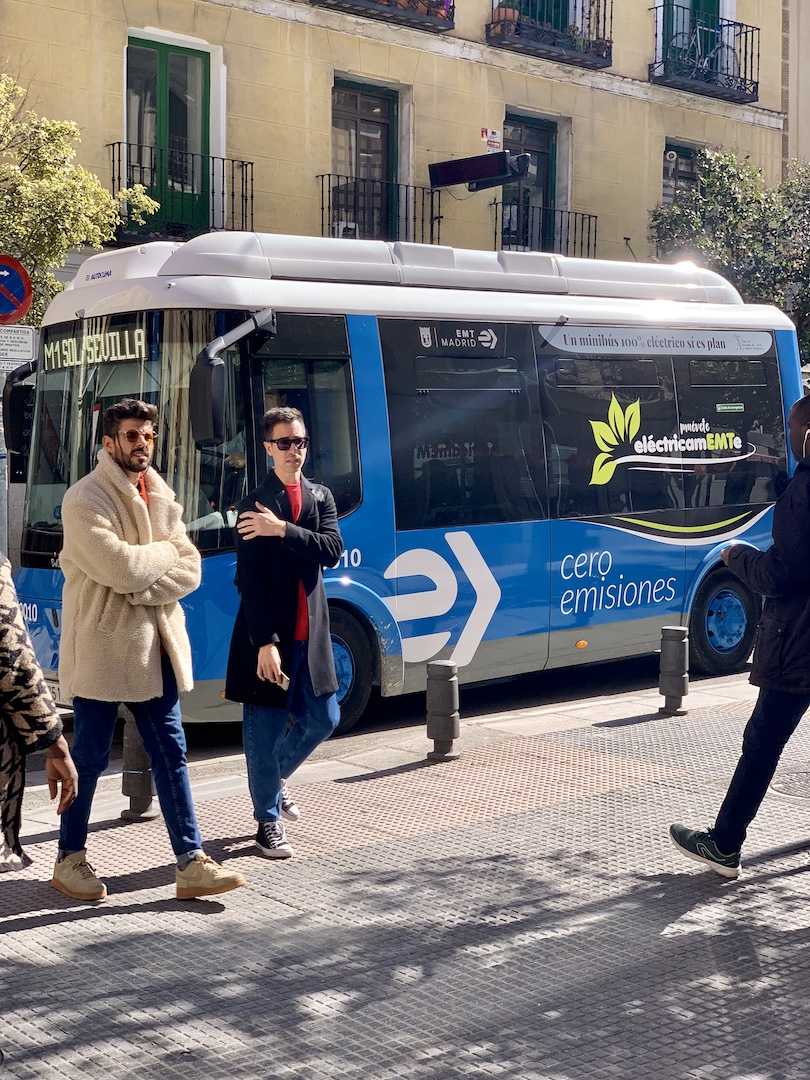

Supplement: Supplemental Information 1 [file peerj-cs-11-2085-s001.zip › ultralytics/assets/bus.jpg]
